# Current concepts in locking plate fixation of proximal humerus fractures

**DOI:** 10.1186/s13018-017-0639-3

**Published:** 2017-09-25

**Authors:** Christoph J. Laux, Florian Grubhofer, Clément M. L. Werner, Hans-Peter Simmen, Georg Osterhoff

**Affiliations:** 1 Department of Trauma Surgery, University Hospital Zurich, Rämistrasse 100, 8091 Zurich, Switzerland; 2 Balgrist University Hospital, Forchstrasse 340, 8008 Zurich, Switzerland

**Keywords:** Proximal humeral fracture, Locking plate fixation, Medial support, Calcar screws, Cement augmentation, Bone graft

## Abstract

Despite numerous available treatment strategies, the management of complex proximal humeral fractures remains demanding. Impaired bone quality and considerable comorbidities pose special challenges in the growing aging population. Complications after operative treatment are frequent, in particular loss of reduction with varus malalignment and subsequent screw cutout. Locking plate fixation has become a standard in stabilizing these fractures, but surgical revision rates of up to 25% stagnate at high levels. Therefore, it seems of utmost importance to select the right treatment for the right patient. This article provides an overview of available classification systems, indications for operative treatment, important pathoanatomic principles, and latest surgical strategies in locking plate fixation. The importance of correct reduction of the medial cortices, the use of calcar screws, augmentation with bone cement, double-plate fixation, and auxiliary intramedullary bone graft stabilization are discussed in detail.

## Background

Due to current demographic changes, the number of proximal humeral fractures will continue rising dramatically, especially in female individuals [1]. Non-operative treatment with short-term immobilization is a well-approved treatment option and has shown good clinical results in stable and minimally displaced fractures as well as in certain displaced fractures. However, conservative therapy regimes are not reserved to simple fractures alone, as the functional outcome primarily depends on age and less on deformity [2, 3]. It is to be expected, though, that particularly complex proximal humeral fractures with severe displacement will occur more often in the aging society with longer life expectancy and notable comorbidities [4]. Despite latest developments of fixation techniques and implants, the surgical treatment of these—mainly osteoporotic—fractures remains challenging. Primary arthroplasty has to be considered in fractures where sufficient reduction and stable fixation cannot be achieved and the vascularity of the head fragment is impaired or at risk. In most remaining cases, especially when displacement of the tuberosities is present, locking plate fixation has proved to be the gold standard. Anterograde intramedullary nailing is often not an option for complex fractures and has the considerable disadvantage of affecting the rotator cuff. Intramedullary locking nails are best applicable in displaced two-part fractures or three- and four-part fractures with meta- or diaphyseal involvement and no significant displacement of the tuberosities [5]. This article aims to summarize available classification systems, indications for operative treatment, important pathoanatomic principles, and latest surgical strategies in locking plate fixation. The importance of correct reduction of the medial cortices, the use of calcar screws, augmentation with bone cement, double-plate fixation, and auxiliary intramedullary bone graft stabilization are discussed in detail.

## Methods

For the arrangement of this narrative non-systematic review, an exploratory search in the MEDLINE database using the keywords “proximal humeral fracture,” “locking plate,” “classification,” and “treatment” was conducted. Clinical and experimental studies were included in a detailed review. In addition, references of reviewed articles were searched for relevant studies not yielded by the initial search.

## Fracture classification

The classification of proximal humerus fractures has always suffered from poor intra- and interobserver reliability [6, 7], especially in plain radiographs. Thus, many classification models have been proposed over the decades. Codman’s illustrative classification system proposed in 1934 is the most influential concept and serves as basis for many classification models in clinical practice. Based on the configuration of the four main segments—namely the humeral head, the shaft, the greater tuberosity, and the lesser tuberosity—12 fracture patterns can be distinguished. Neer’s analysis of 300 displaced proximal humeral fractures in 1970 led to a modification with additional subgroups and focus on the pathoanatomy regarding presence or absence of displacement of the four bony segments rather than fracture lines [8]. The definition of displacement was “arbitrarily set” and requires at least 1 cm of separation and 45° of angulation between fragments [9]. As displacement is a continuum, he further clarified that these limits were not intended to dictate treatment but simply to define the minimal displacement category (1-part) and to support standardization in future outcome studies [9]. Neer was not surprised about the poor reproducibility of his classification system in radiologic studies as a pathoanatomic classification may even require intraoperative assessment. Although the accuracy in its application often has been questioned [6, 10–12], Neer’s classification still enjoys broad acceptance and common use.

Hertel et al. introduced a further development of Codman’s concept taking the fracture planes but not the number of fragments into account [13]. Combining the five basic fracture planes (between greater tuberosity and head, greater tuberosity and shaft, lesser tuberosity and head, lesser tuberosity and shaft, and between lesser and greater tuberosity) yields 12 basic fracture patterns. More precisely, there are six possible fractures dividing the humerus into two fragments, five possible fractures dividing the humerus into three fragments, and a single fracture dividing the humerus into four fragments [7, 13]. When the dichotomizing questionnaire for determining the fracture morphology could not be certainly answered on plain radiographs, additional imaging studies (computed and/or magnetic resonance tomography) should be demanded. The evaluation of seven accessory questions provides important information for further treatment planning, especially on the probability of humeral head ischemia (Table 1). Compared to the other available classification systems, the *κ* coefficient for interobserver reliability ranked highest in the Codman-Hertel classification [7].

**Table 1:** Predictors of humeral head ischemia after intracapsular fracture of the proximal humerus [13]

Predictors of posttraumatic humeral head ischemia	
• Metaphyseal extension < 8 mm • Disrupted medial hinge • Basic fracture morphology • Head-split component (> 20% head involvement) • Angular head displacement > 45° • Tuberosity displacement > 10 mm • Glenohumeral dislocation	

Despite recent modifications, the AO classification system for proximal humerus fractures plays a rather scientific role and—in contrast to other fracture sites—has not found its way into clinical use due to its complexity with a total of 27 subtypes [14, 15].

As a supplement to the Codman-Hertel classification system, Resch proposed a classification addressing fracture angulation and pathomechanics [16]. It further evaluates the head-to-shaft relationship and, thus, is supposed to facilitate reduction and fixation during surgery.

## Indications for operative treatment

The heterogeneity of proximal humerus fractures not only complicates the search for a reproducible classification system but also—as a consequence of poor comparability—delays the definition of coherent treatment protocols. Despite the frequency of proximal humerus fractures, until now, there is no solid evidence on treatment indications [17, 18].

Absolute indications for an operative treatment of proximal humerus fractures are rare. These comprise three- or four-part fracture dislocations, head-splitting fractures, pathological fractures, open fractures, severe ipsilateral injuries to the shoulder girdle, and accompanying neurovascular injuries [17, 18]. However, with a displacement greater than 5 mm, reduction and internal fixation is recommended as secondary salvage surgery after failed non-operative treatment with a corrective osteotomy or secondary arthroplasty is more difficult and less promising [19].

Along with the fracture pattern, patient age, and overall state, comorbidities and associated medication, handedness, and expected demands on the injured extremity have to be taken into account. If the bone quality is unknown, the deltoid tuberosity index is a simple tool to estimate the bone quality in an anterior-posterior radiograph [20]. Especially in geriatric patients, close cooperation with a geriatric physician is advisable to facilitate early active rehabilitation after operative treatment [21].

In unreconstructable humeral head fractures, head-split fractures or fracture dislocations, and patients older than 70 years with high risk of osteonecrosis or previously impaired shoulder, function primary (reverse or anatomic) arthroplasty may be the best therapeutic option. This also includes patients with delayed presentation and glenoid damage or wear [18, 22].

## Non-operative treatment

Despite the risk of nonunion, symptomatic malunion or osteonecrosis, non-operative therapy even of complex proximal humerus fractures may be adequate in the very elderly or cognitive impaired population and in patients with a nonfunctional limb, well advanced drug or alcohol abuse or severe medical comorbidities [17]. These settings often require close cooperation with a geriatric physician in order to prevent secondary conditions and further falls. Non-operative treatment usually comprises a short interval of sling immobilization (i.e., 3 weeks) and subsequent early pendulum exercises, followed by active rehabilitation to restore shoulder function and achieve independency [23, 24].

Non-displaced one- and two-part fractures typically are treated conservatively and thereupon yield excellent radiographic union rates and good functional range of motion [25, 26]. However, age has been found to be a predictor of impaired outcome in this patient group [27].

Serial radiographs are necessary to monitor the course of treatment. In order to obtain comparable radiographs, it is important to appreciate a proper arm positioning. Sling immobilization puts the arm in internal rotation, which misleadingly increases the head-shaft angle and simulates a valgus malalignment [28]. Thus, neutral arm with the palm of hand on the lateral thigh is crucial for initial and follow-up examinations. The angulation on Y view best correlates with the functional outcome and best predicts the angulation at union [29, 30].

## Principles of operative treatment

### Head ischemia

Avascular necrosis of the humeral head is a known sequela of proximal humerus fractures and occurs at rates of 3 to 68% [31–33]. The humeral head is mainly perfused by the ascending branch of the anterior humeral circumflex artery, also known as the arcuate artery as it subchondrally traverses the entire humeral head in an arch-shaped manner [34]. However, the posterior humeral circumflex artery also considerably contributes to the blood supply of the humeral head as it dispatches distinctive branches during its dorsal course around the surgical neck [35]. As shown in cadaveric studies, these branches gain crucial importance in a setting of posttraumatic head perfusion [36]. Regardless of the chosen treatment option, fractures of the anatomical neck are prone to avascular necrosis of the head fragment due to disruption of the dominant nutrient artery. If, however, the medial extension of head fragment reaches far enough distal to the articular surface, some perfusion persists by means of the posteromedial vessels. In terms of a sufficient residual head perfusion, the least posteromedial metaphyseal extension of the head fragment has been numbered 8 mm according to Hertel et al. [37]. They found ischemic humeral heads to rather have a disrupted medial hinge with a shaft displacement greater than 2 mm in any direction. Moderate and poor predictors for head ischemia were basic fracture type, angular head displacement greater than 45°, tuberosity displacement greater than 10 mm, glenohumeral dislocation, and head-split components. In combination, these criteria (anatomic neck fracture, short calcar segment, and disrupted hinge) yielded positive predictive values of up to 97% [13].

### Reduction

In osteoporotic bone, reduction might be difficult to obtain and yet—independent of the chosen implant—precise anatomic reduction is the cornerstone of a stable fixation and essentially enhances its longevity [38]. Therefore, correct interpretation of the fracture pattern and its trauma mechanism is essential. Knowledge of the deforming forces of the muscular attachments very much helps in reducing and retaining displaced fractures. As to Codman et al., the main fragments consist of the major and minor tubercle, the humeral head, and the shaft. The displacing forces of the attaching muscles lead to a medial displacement of the shaft due to the pull of the pectoralis major muscle and to an external rotation and varus angulation of the head fragment or the separated tubercular fragments along the muscle pull of the rotator cuff. The humeral head or the articular fragment can also be pushed into a valgus deformity due to the axial load of the trauma.

First and foremost, the integrity of the medial hinge—the so-called calcar—must be ascertained and in case of disruption reconstructed before further reduction maneuvers are applied. The most efficient method to gain osseous medial support of the humeral bone is perfect reduction of the medial cortices. The medial periosteum plays a key role in the fracture management, because it allows indirect reduction using ligamentotaxis and it maintains the blood supply of the head fragment via branches of the posterior humeral circumflex artery.

Krappinger and colleagues postulated that anatomical fracture reduction and the correct alignment of the medial cortices are the two most important prognostic factors in terms of secondary displacement [39, 40]. Because of neighboring neurovascular structures and the insertion of rotator cuff and biceps tendons, extra-medullary fixation of proximal humeral fractures mostly has to be approached from the lateral aspect [34, 35]. Therefore, reduction of the medial fracture zone can only be achieved through indirect manipulation or across the fracture line. Direct visual control is not possible. To confirm perfect reduction fluoroscopy is mandatory. Fractures with medial comminution are technically difficult or not at all manageable. In some cases, the treatment of choice then is the intended impaction of the humeral head.

Biomechanical studies could prove that even with correct axial reduction, missing calcar stabilization leads to secondary displacement with varus impaction of the humeral head [41].

### Medial support

Advanced biomechanical research has shown that the medial osseous stability of the humerus is an essential prerequisite for a satisfactory functional outcome of patients with proximal humeral fractures.

The loss of medial support is the most common reason for secondary displacement with varus impaction [39, 42]. Varus displacement of 20° already significantly elevates the forces of the rotator cuff for elevation movement [43], thus severely limiting the functional outcome [42]. Due to the increased rigidity of locking plate systems [38], dislocation and even penetration—or “cutout”—of the locking screws, especially in fractures with varus impaction, is reported frequently and can cause severe cartilage damage of the glenoid cavity. Gardner and colleagues showed that loss of medial support results in a fivefold higher cutout rate of the locking screws. In their analysis, a fixation is considered to provide adequate medial support if either the medial cortex is intact anatomically reduced, and not comminuted or there is a stable head-on-shaft impaction or a superiorly directed oblique locking screw is appropriately placed into the inferomedial quadrant of the proximal humeral head fragment [39].

## Implants and surgical techniques

### Locking plate

The inadequate implant anchorage in osteoporotic bone is a major issue, which inhibits a sufficient and stable osteosynthesis. In order to address insufficient screw purchase in conventional plate fixation, locking plate systems have been developed combining rotational and angular stability with higher resistance to failure [44]. These fixation systems are able to stabilize fracture fragments without friction between plate and bone [45] and thus provide more stability in osteoporotic bone [46]. Despite remarkable functional results, complication rates remain high [32, 42, 45, 47, 48]. A major reason for secondary displacement is the low bone quality, the stiffness of the implant, and the high peak stress at the bone-implant interface.

Especially in fractures with medial comminution, the following principles have become important in order to increase the stability of locking plate fixation of proximal humerus fractures.

### Calcar screws

Gardner et al. suggested obliquely positioned inferomedial screws as an additive support tool. A calcar screw reduces the risk of a varus collapse with subsequent screw perforation by counteracting the varus deforming forces acting on the humeral head. This results in a significantly higher reposition stability after 6 and 12 months [39, 40] and increases the failure load [49].

With new minimally invasive techniques, the need for calcar screws often has been questioned. However, the positive clinical impact of calcar screws in terms of complication rate, fracture reduction, and Constant score has been repeatedly shown, especially for more complex fractures [50, 51]. In order not to harm the axillary nerve in minimal invasive plate osteosynthesis, the insertion of calcar screws should only be performed under direct vision [52]. The insertion of calcar screws does not increase the risk of humeral head necrosis by compromising the medial periosteal blood supply [53]. Insertion of more than one calcar screw does not provide additional torsional or axial stability [54]. A proximal screw perforation is seen in 6–8% of patients treated with calcar screws [39, 40].

### Cement augmentation

Especially in patients with low bone mineral density, stable implant anchorage is difficult. In addition, shear forces at the bone-implant interface favor loss of reduction after locking plate fixation. Screw augmentation with bone cement (polymethyl methacrylate, PMMA) significantly improves the primary stability [55, 56] and reduces the motion at the bone-implant interface. Concerns of a critical temperature increase due to the exothermic reaction of PMMA resulting in necrosis and subsequent implant loosening do not seem justified. In an analysis by Blazejak et al., the threshold values for necrosis and apoptosis of cartilage and subchondral bone provided in the literature have not been reached [57]. Thus, in patients with impaired bone mineral density, cement augmentation either directly to the head prior to screw insertion or via cannulated and perforated screws can be a valid option to decrease the risk of varus impaction and is already applied in clinical practice [58].

### Double-plate fixation

A few authors suggest a gain of medial stability through the additional use of one-third tubular plates positioned ventral and right-angled to the lateral adjusted standard plate [59]. This procedure leads to less biomechanical stability compared to the osteosynthesis with locking plates systems [60]. The ventral inserted plate is able to harm the blood support of the arcuate artery, which is a branch of the anterior humeral circumflex artery [35].

In a case series published in 2011, four patients were treated with lateral locking plate systems and an intramedullary inserted one-third tubular plate [61]. The challenges in case of revision or secondary joint replacement will be seen in future.

### Bone grafting

**Fig. 1:**
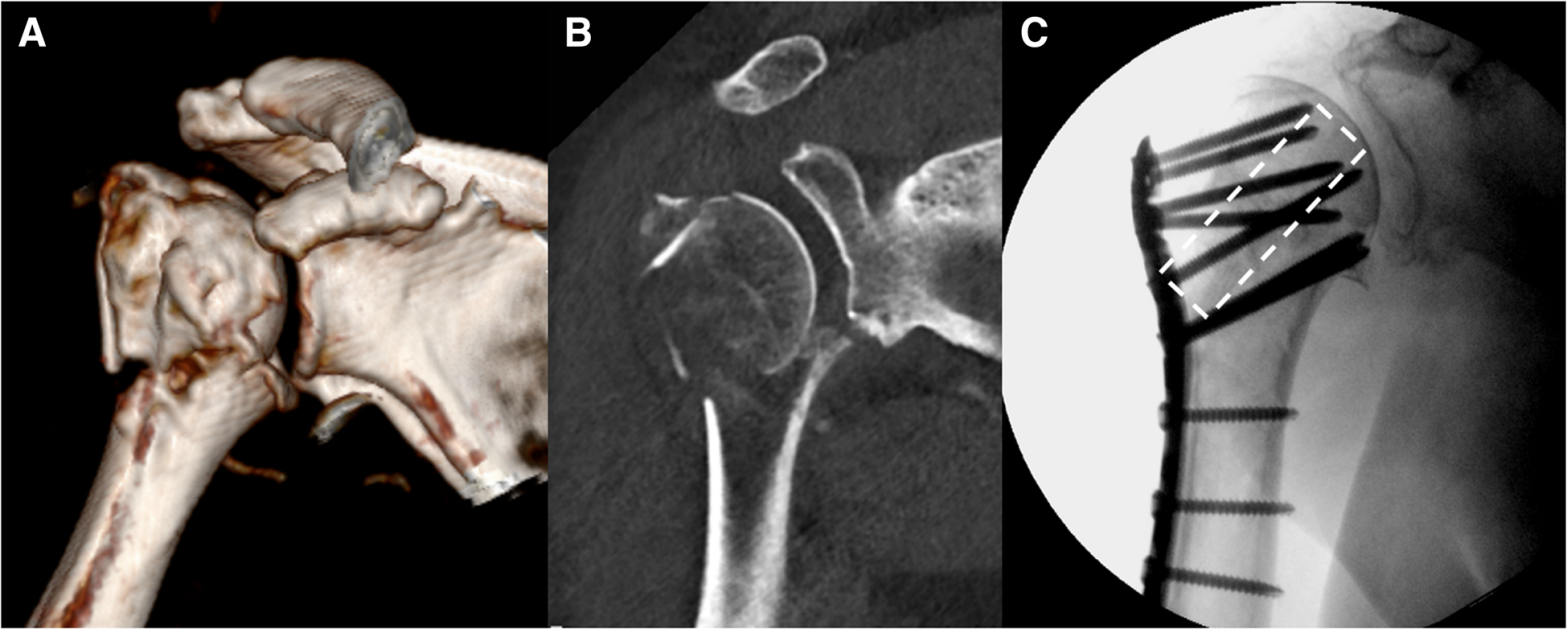
Case of a 71-year-old female patient with a proximal humerus 3-part fracture with an impacted articular fragment (**a**, **b**). Due to the substantial metaphyseal loss of trabecular bone matrix, a fibula allograft (dashed outline) was used to support the locking plate fixation construct (**c**)

If the reduction of a comminuted calcar area cannot be achieved, a locking plate system combined with a corticocancellous bone graft can be considered (Fig. 1). In a first case series, allogenic fibular grafts were used in a patient group with medially bruised calcar. After 6 months, no secondary loss of reduction was seen [62].

The authors could prove the biomechanical advantages of this technique in an in vitro study with synthetic bone [63]. Standardized proximal humerus fractures were created in a synthetic osteoporotic bone. In one group, osteosynthesis was performed with a lateral locking plate combined with calcar screws. In the second group, an intramedullary fibular graft of 6-cm length supplemented the lateral locking plate. Active abduction was simulated for 400 cycles by use of an established testing setup. The measurements verified a five times lower range of intercyclic motion, a 50% reduction of fragment migration, and a 50% reduction of plastic deformity of the intramedullary graft model group.

These results are in concordance with cadaver studies, where an additional bone graft increased stiffness and varus failure load of the locking plate-bone complex [54, 64, 65].

### Other techniques

Shoulder arthroplasty is a well-established therapy option in case of a complex humerus fracture in the elderly [66–68]. The rotator cuff tendons and the greater and lesser tuberosities are often traumatically compromised and do not heal adequately to the prosthetic component. These concomitant damages and nonunions explain the higher failure rate of hemiarthroplasties in comparison with reverse total shoulder arthroplasties (RTSA) in proximal humeral fractures [69]. In this patient group, reverse shoulder arthroplasty seems to be the preferable strategy when compared with hemiarthroplasty [70, 71]. RTSA restores an acceptable shoulder function with high patient satisfaction rates even if irreparable rotator cuff damages are present [67, 72]. The RTSA design as proposed by Grammont bases on medialization of the center of rotation, which results in a greater lever arm of the deltoid muscle [73]. The medialization of the rotational center implicates also lesser tension of the rotator cuff muscles on the fractured tuberosities, which might diminish the risk of secondary dislocations and make a more practicable rehabilitation—especially for elderly patients—possible. RTSA also seems to be a reliable and good salvage option if primary osteosynthesis of the proximal humerus has failed [74].

Intramedullary nailing also is a broadly used fixation method in proximal humerus fractures. However, it could not be proved superior when compared to locking plate fixation [75]. In their meta-analysis, Wang et al. only found limited evidence without significant difference in terms of clinical outcome suggesting that locking plate and intramedullary nail both are valuable options for the treatment of proximal humeral fractures [76]. A recent randomized controlled trial by Gracitelli et al. analyzing 65 patients also yielded similar Constant-Murley and Disability of the Arm, Shoulder, and Hand (DASH) scores and equivalent neck-shaft angles after 12 months [77]. However, a significantly higher complication and reoperation rate was observed with intramedullary nailing. Shoulder pain is a well-documented complication of antegrade humeral nailing that is considered technique-specific due to iatrogenic rotator cuff damage during nail preparation and insertion [78, 79]. A more medial insertion through the supraspinatus muscle belly therefore is proposed [79] but puts the articular cartilage at risk.

### Surgical approach

The anterior deltopectoral approach with the patient in beach-chair position clearly is the working horse in the surgical treatment of proximal humerus fractures. The biceps tendon serves as important landmark when identifying the main fragments but also when assessing the reduction [80]. If required, the articular surface can be examined via a small incision of the rotator interval. The deltopectoral approach also provides valuable options for extension both distally and proximally. With these measures, fractures involving the humeral shaft can be addressed and bleedings of the axillary artery can be controlled. Furthermore, the anterior approach can be used in later arthroplasty. Special attention should be paid to the musculocutaneous nerve entering the body of the coracobrachialis muscle (5 to 8 cm distal to the muscle origin) and the cephalic vein in the deltopectoral groove [81].

The aforementioned transdeltoid lateral approach only provides limited access to the humeral head but can be used for osteosynthesis of the greater tuberosity or minimally invasive plate osteosynthesis (MIPO) of the proximal humerus. The axillary nerve transverses the deltoid muscle about 7 cm below the tip of the acromion, and thus, the incision should not exceed 5 cm starting at the tip of the acromion [52]. The nerve can easily be palpated as on the deep surface of the deltoid muscle [81]. For MIPO, the approach is expanded by additional stab incisions safely below the axillary nerve.

The posterior approach uses a linear incision along the scapular spine and the internervous plane between infraspinatus and teres minor. It might be needed in the treatment of posterior fracture dislocation or when addressing concomitant scapular fractures.

## Discussion

The locking plate osteosynthesis of displaced proximal humerus fractures remains a challenge for the upper extremity surgeon. Despite development of new implants and awareness of new biomechanical fracture characteristics, the complication rate stagnates on a high level.

Especially varus impaction with penetration of proximal screws is a frequent complication [32, 42, 82, 83]. Even in cases of anatomic reduction and the use of calcar screws, in 6 to 8% of the patients a screw cutout is seen [39, 53]. In general, complications occur within the first 3 weeks after surgery, when patients start physical therapy [84]. However, for monitoring the vitality of the humeral head, a follow-up over 2 years seems appropriate.

In view of these severe complications, most proximal humerus fractures can be treated non-operatively. However, in selected patients with fractures with relevant intraarticular damage or displacement and after failed non-operative treatment, operative treatment is the preferred strategy to improve the patient’s functional outcome. In these patients, locking plate fixation provides an established mode of fixation.

To reduce peak stresses at the bone-implant interface that lead to screw cutout and early loosening, the ideal implant needs to provide elastic characteristics [38]. However, the initial stiffness of locking plates is needed for stability especially in osteoporotic bone—the higher the stability, the faster the bone healing [85]. In our group’s biomechanical in vitro study, the combination of locking plate osteosynthesis and intramedullary cortical bone graft seems to have met these opposing demands [63].

Neviaser et al. delivered the clinical evidence of this method in a case series of 34 patients with intramedullary fibula grafts [61]. In this series, only one patient presented secondary displacement but did not need revision surgery. The appearance of only one patient with partial humeral head necrosis defuses the fear of humeral head necrosis caused by compromised intramedullary blood supply. To avoid ischemic humeral head necrosis, precise anatomic knowledge of the posteromedial periosteal blood support and a careful surgical dissection is an essential demand.

The posterior circumflex humeral artery covers two third of the proximal humeral blood supply [44]. In proximal humerus fractures, this artery remains the last supplying vessel. Uncontrolled shear forces between the humeral diaphysis and the humeral head need to be avoided. The disruption of the posteromedial periosteum appears with a head displacement of about 3 mm. The complete disruption is seen with an average displacement of 30 mm.

In summary, the intramedullary bone grafting should be reserved for osteoporotic proximal humerus fractures with a significant displacement of the humeral head and medial comminution. The economic costs of allogenic bone grafts or the comorbidities of the autologous bone grafting technique, respectively, should be regarded critically.

Augmented osteosynthesis with bone cement should also be mentioned as a treatment option, although removal of the incorporated cement represents considerable disadvantages in case of secondary prosthetic joint replacement.

Retrograde intramedullary nailing is a new stabilization approach. Dietz et al. compared retrograde nailing versus locking plate systems as treatment option for two-part proximal humeral fractures. They could not find any differences in stability for axial and torsional loading [86].

Especially in elderly patients with humeral head fractures with high risk of osteonecrosis or previously impaired shoulder function, primary shoulder arthroplasty may be the best therapeutic option. Reverse total shoulder arthroplasty shows reliably good results with relatively low complication rates compared to osteosynthesis in the same patient population [69]. Hemiarthroplasty fails if concomitant irreparable rotator cuff damages or non-malunion of the tuberosities are present.

## Conclusion

The treatment of proximal humerus fractures remains challenging. When the decision for surgical fixation is made, anatomic reduction with restoration of medial support and protection of vascular and periosteal structures are crucial prognostic factors and the most reliable feature in the prevention of secondary varus dislocation. Locking plate fixation offers a widely employable fixation method that can be enhanced with calcar screw cement augmentation or bone grafts in case of comminuted fractures. In geriatric patients, the treatment often is non-operative or with reverse total shoulder arthroplasty as the complication rate of osteosynthesis in the elderly is high. A close cooperation with a geriatric physician is recommended for the purpose of early active rehabilitation and to prevent secondary conditions.
